# Are youth sport talent identification and development systems necessary and healthy?

**DOI:** 10.1186/s40798-018-0135-2

**Published:** 2018-05-22

**Authors:** Fieke Rongen, Jim McKenna, Stephen Cobley, Kevin Till

**Affiliations:** 1 Institute for Sport, Physical Activity and Leisure, Leeds Beckett University, Leeds, UK; 2 Discipline of Exercise & Sport Science, Faculty of Health Sciences, The University of Sydney, Sydney, Australia; 3 Yorkshire Carnegie RUFC, Leeds, UK; 4 Leeds Rhinos RLFC, Leeds, UK

**Keywords:** Talent identification, Talent development, Health, Adolescence, Impact

## Abstract

Talent identification and development systems (TIDS) are commonly used in professional sport to convert youth athletes into sporting stars of the future. Acknowledging that only a few athletes can “make it,” the necessity and healthiness of TIDS have recently been questioned based on their increased professionalism, high training, and competition volumes, but limited effectiveness. In this short communication, we suggest that the key issues associated with TIDS are not due to their overall concept, but with how they are designed and implemented. It is recommended that researchers and practitioners determine the worth and value of TIDS by also evaluating the positive health of the athlete rather than solely focusing on performance outcomes. To achieve this, TIDS staff should shape and develop their values, expectations, and day-to-day routines to achieve positive health outcomes focusing on personal development and an athlete-centered culture. In business, this has been termed the concept of “Deliberately Developmental Organisation.” TIDS can deploy the factors (e.g., high-quality staff, expert support services, quality facilities, and learning routines) characteristic of such organizations, to concurrently ensure positive impacts and minimize predictable negative outcomes without losing focus on a drive for sporting performance.

## Key points

- Talent identification and development systems (TIDS), although aimed at sporting performance, impact upon physical health, education, and psycho-social development within youth athletes.
- The deployment of TIDS influences whether these impacts are positive or negative, and (un)intended. As only limited numbers of athletes can achieve elite sport success, TIDS effectiveness should encapsulate these impacts and staff should emphasize these impacts to ensure system “worth.”
- TIDS staff can utilize a “Deliberate Development Organisation” approach to emphasize an athlete-centered culture with high-quality staff, expert support services, and quality facilities to maximize positive outcomes in their athletes.

## Introduction

Globally, talent identification and development systems (TIDS) have proliferated [1]. Given their increasing professionalism and intensity, TIDS are resource-intensive requiring substantial financial investment. To recover these costs, TIDS are expected to be effective in converting youth athletes into medal winning or saleable assets within adult sports [2]. Recently, TIDS across a range of sports have been questioned in the research literature for their effectiveness [3, 4] and healthiness (e.g., injury, psychological overload; [5–7]). In England’s soccer-based academies, this approach to TIDS has recently been criticized by the media [8, 9], suggesting endemically inadequate support for young boys and men, under the auspices of the Elite Player Performance Plan (EPPP).

Such issues raise concerns about the necessity and healthiness of TIDS, which in soccer alone, through the EPPP, engage ~ 12,000 boys and young men in intensive training four to five times per week [10, 11]. Due to the exclusive nature of elite sport, at face value, these programmes offer participants little likelihood of securing elite sporting “success” as adults [12]. The requirement of being released from school to train can also restrict educational development and attainment, while the scale of mental ill-health recently reported in ex-academy players clearly questions the EPPP aims of supporting “the holistic development of young players” and of developing “a well-rounded player.”

## Moving towards impact

TIDS are often pyramidally structured, suggesting that limited system effectiveness is an expectation; as such, we suggest that evaluating TIDS against their impact may offer more valuable insights [1]. Impact here does not ignore sporting performance but encapsulates overall youth development. For example, due to the depth of involvement that TIDS mandate, it is unsurprising that physical health, educational and social life, identity, and psycho-social development are all impacted upon in (un)intended and positive and/or negative ways.

Taking “positive health” as a focus, TIDS have three domains of impact: (1) subjective (e.g., feeling great), (2) biological (physiological capacity), and (3) functional (being productive in daily life) [13]. TIDS athletes often report impact in these areas, including physical adaptations linked to the need for increased physiological capacity vs. the management of training volume, intensity, and injury. Recently, health outcomes have been acknowledged in new consensus statements (e.g., IOC statement on training load and injury [14]) and football-specific literature [11, 15]. There are parallel tensions around balancing positive and negative subjective (e.g., psychological, social) and functional (e.g., educational) outcomes, yet these have rarely been examined and evaluated, suggesting the benefits of further investigation.

## Moving forward

Although recent research evidence [5–7] suggests something amiss, and this account may seem downbeat, contemporary evidence suggests that important positive health and well-being impacts emerge from higher quality TIDS [16]. Therefore, our suggestion is that key issues do not lie with the overall concept of TIDS; they are neither inherently good nor bad. Instead, impact reflects how well they are designed, implemented, and managed so that youth athletes systematically secure positive health outcomes. Given that behavioral design is such a contemporary issue (e.g., [17]), it is logical to expect that positive health constructs can be used to develop TIDS that simultaneously balance multiple training (e.g., load), psychological (e.g., identity, autonomy) and social (e.g., sense of belonging) contexts, and cultural (e.g., values) factors that, otherwise, can be especially challenging for youths [1].

While acknowledging the importance of focused, progressively intensive deliberate practice in athlete development, it is likewise important to pay closer attention to managing the experiential “diet” of, and within, individual TIDS. The values, expectations, and day-to-day routines that characterize experience within any TIDS will all shape the emergence of positive and/or negative impacts. In business, the concept of a “Deliberately Developmental Organisation” (DDO) applies to fewer than 3% of top-level organizations [18], suggesting that what we are proposing will not be found in every TIDS. DDOs represent organizations embracing a belief that people’s strongest motivation in professional environments is to grow personally and professionally [18]. Companies prosper in terms of both economic returns and employee satisfaction and health when they use deliberate practice principles to create the conditions for growth to happen, “fashioning an organisational culture in which support of people’s development is woven into the daily fabric of working life and the company’s regular operations, daily routines, and conversations” ([18] p. i). rather than quick fix solutions (e.g., continued development courses and development days).

The strength of any DDO lies in consistently and coherently connecting its values (i.e., focus on personal growth) to everyday actions, routines, and processes. DDOs are successfully driven by staff who align their day-to-day routines and practices to not only deliver key business outcomes but also prioritize the personal development of individuals. Given the problems experienced by young men with an over-developed athletic identity, this seems an immediate personal development priority. In sport, the nearest equivalent to a DDO is the focus on delivering an “athlete-centered” culture that focuses on concurrently building “responsible autonomy” alongside sporting performance. This approach requires well-trained staff who create and deliver developmentally appropriate challenges while building caring and authentic relationships with their athletes.

## Conclusion

**Fig. 1:**
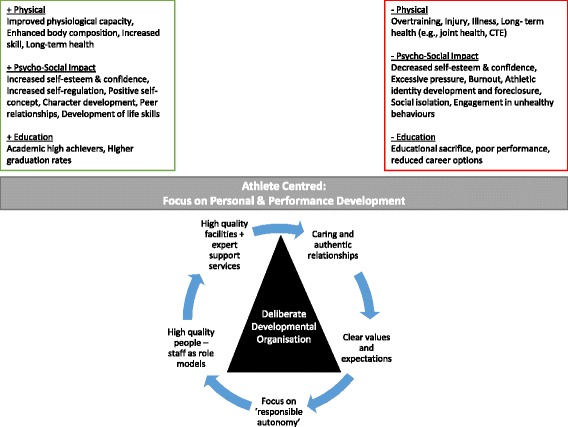
Deliberate developmental organization for balancing youth athlete health within talent identification and development systems

While TIDS are now common in youth sport, recent speculation has questioned their necessity and healthiness. Recognizing that only few athletes can “make it” within elite sport, it is now imperative to assess TIDS against their impact on the many, rather than their effectiveness in producing the few. With DDOs, a range of factors can be deployed (e.g., high-quality staff, expert support services, quality facilities and learning routines), to ensure positive impacts and minimize predictable negative outcomes (see Fig. 1). It is timely to expect that TIDS are evaluated for program impact and their ability to optimize targeted features of both athlete and personal development.
